# Evaluating intra‐fractional tumor motion in lung stereotactic radiotherapy with deep inspiration breath‐hold

**DOI:** 10.1002/acm2.14414

**Published:** 2024-05-27

**Authors:** Weihua Fu, Yongqian Zhang, Kiran Mehta, Alex Chen, Hima Bindu Musunuru, Pietro Pucci, Jason Kubis, M. Saiful Huq

**Affiliations:** ^1^ Department of Radiation Oncology University of Pittsburgh School of Medicine and UPMC Hillman Cancer Center Pittsburgh Pennsylvania USA

**Keywords:** deep inspiration breath‐hold, DIBH, intra‐fractional motion, SBRT, stereotactic body radiotherapy

## Abstract

**Purpose:**

To evaluate the intra‐fractional tumor motion in lung stereotactic body radiotherapy (SBRT) with deep inspiration breath‐hold (DIBH), and to investigate the adequacy of the current planning target volume (PTV) margins.

**Methods:**

Twenty‐eight lung SBRT patients with DIBH were selected in this study. Among the lesions, twenty‐three were at right or left lower lobe, two at right middle lobe, and three at right or left upper lobe. Post‐treatment gated cone‐beam computed tomography (CBCT) was acquired to quantify the intra‐fractional tumor shift at each treatment. These obtained shifts were then used to calculate the required PTV margin, which was compared with the current applied margin of 5 mm margin in anterior‐posterior (AP) and right‐left (RL) directions and 8 mm in superior‐inferior (SI) direction. The beam delivery time was prolonged with DIBH. The actual beam delivery time with DIBH (*T*_beam_DIBH_) was compared with the beam delivery time without DIBH (*T*_beam_wo_DIBH_) for the corresponding SBRT plan.

**Results:**

A total of 113 treatments were analyzed. At six treatments (5.3%), the shifts exceeded the tolerance defined by the current PTV margin. The average shifts were 0.0 ± 1.9 mm, 0.1±1.5 mm, and ‐0.5 ± 3.7 mm in AP, RL, and SI directions, respectively. The required PTV margins were determined to be 4.5, 3.9, and 7.4 mm in AP, RL, and SI directions, respectively. The average *T*_beam_wo_DIBH_ and *T*_beam_DIBH_ were 2.4 ± 0.4 min and 3.6 ± 1.5 min, respectively. The average treatment slot for lung SBRT with DIBH was 25.3 ± 7.9 min.

**Conclusion:**

Intra‐fractional tumor motion is the predominant source of treatment uncertainties in CBCT‐guided lung SBRT with DIBH. The required PTV margin should be determined based on data specific to each institute, considering different techniques and populations. Our data indicate that our current applied PTV margin is adequate, and it is possible to reduce further in the RL direction. The time increase of *T*_beam_DIBH_, relative to the treatment slot, is not clinically significant.

## 1 INTRODUCTION

Stereotactic body radiation therapy (SBRT) has played a crucial role in lung cancer treatment and shown efficient local control according to reported outcomes.^1^, ^2^, ^3^, ^4^ The success of lung SBRT relies on the accuracy of treatment delivery. The respiratory motion of the lung tumor poses a significant challenge in SBRT and is considered one of the largest sources of uncertainty. Therefore, effective motion management is vital in lung SBRT.

Motion management strategies^5^, ^6^ in lung SBRT include internal target margin covering the full range of motion, respiratory gating, breath‐hold, and tumor tracking. However, the use of a large internal margin in the presence of significant motion may result in unnecessary irradiation of surrounding normal tissue or critical organs, potentially increasing complications. Tumor tracking, offering real‐time adjustments to the radiation beam, ensures optimal radiation doses to the targeted tumor while minimizing exposure to healthy surrounding tissues. However, this method can be technically complex and may necessitate sophisticated equipment. Presently, it is not widely available in most clinics.

Respiratory gating and breath‐hold are the most employed motion mitigation methods in clinical settings. However, in cases of substantial target motion, the gating window may narrow, potentially leading to prolonged treatment times. Breath‐hold proves advantageous^7^, ^8^, ^9^, ^10^ in better sparing surrounding normal tissue or organs at risk (OARs) by reducing planning target volume (PTV) margins, expanding lung volume, or increasing the distance between tumor and OARs. However, successful implementation of breath‐hold relies on the patient's ability to consistently hold their breath during treatment, which may be challenging for some individuals. The choice between motion management strategies needs a careful consideration of patient‐specific factors and treatment objectives.

Deep inspiration breath‐hold (DIBH) treatment has demonstrated benefits^9^, ^11^, ^12^, ^13^ in lung cancer radiotherapy by mitigating respiratory motion and reducing doses to healthy tissues and OARs. The application of advanced treatment techniques, such as volumetric modulated arc therapy (VMAT) and higher dose rate flattening filter‐free (FFF) beam, has contributed to a reduction in treatment delivery time. When combined with hypofractionation of three to five treatments, DIBH becomes a tolerable option for specific patients undergoing lung SBRT.^14^

Presently, image‐guided radiotherapy (IGRT) utilizing kilovoltage cone‐beam computed tomography (CBCT) is widely utilized. This approach provides 3D information for soft tissues, contributing to a substantial improvement in the precision of radiotherapy. With daily pre‐treatment CBCT, the inter‐fractional tumor position variation can be minimized. However, during the beam delivery with DIBH, motion still occurs both during breath‐hold and between consecutive breath‐holds, causing the intra‐fractional tumor position variation. Some studies^15^, ^16^, ^17^ showed small intra‐fractional tumor motion, while Ottosson et al.^18^ observed large intra‐fractional tumor position variations in DIBH lung SBRT. The conflicting findings suggest that the intra‐fractional tumor motion during DIBH may vary based on implementation procedures for different patient populations in different institutions.

This study aimed to quantify the intra‐fractional tumor position variation at our clinic by utilizing CBCTs acquired post‐treatment for patients receiving lung SBRT with DIBH and to investigate the adequacy of the current applied PTV margins.

## 2 METHODS

### 2.1 Patient selection

Twenty‐eight lung SBRT patients with DIBH respiratory motion control were selected in this study. Among these lesions, 23 were in the right or left lower lobe, two in the right middle lobe, and three in the right or left upper lobe in proximity to critical OARs, such as great vessel, esophagus, and trachea. DIBH respiratory motion control technique was used based on the following two conditions.

1) The target motion was large, and if the gating technique were employed, the gating window would be small, potentially leading to prolonged treatment times. In cases where the patient could hold their breath effectively, using DIBH would shorten the treatment time compared to using gating. For instance, one of the left lower target motions measured as 4 cm. If a gating approach were utilized, the gating window would be 40%—70%, resulting in treatment delivery during the 30% of the respiratory cycle and extending the treatment duration. However, given the patient's ability to hold their breath well, DIBH would offer a more efficient alternative.
2) The target was close to OARs. Utilizing DIBH would increase the space between the target and OARs, thereby improving OAR sparing. For instance, a target in the left upper lobe was only 2 mm away from the great vessel (GV). Despite the target motion being less than 5 mm, the internal target volume (ITV) would overlap with the GV if encompassing the entire tumor motion trajectory. With DIBH, the space between the target and GV was increased to 5 mm, resulting in better sparing of the OAR.

### 2.2 Simulation

Patients were positioned supine with arms up and immobilized using the Elekta BodyFix system (Elekta, Stockholm, Sweden). The vacuum blue bag conforms to the patient's body shape, and a vacuum plastic sheet tightly covers the patient, providing additional immobilization and reducing respiratory motion amplitude.

Helical CT and 4D CT images were initially obtained using a GE LightSpeed RT^15^ CT scanner (GE Medical, Milwaukee, WI). Respiratory motion was monitored using Varian Respiratory Gating for Scanners (RGSC) (Varian Medical Systems, Palo Alto, USA). The RGSC employs an infrared‐emitter camera to record the position of a reflective marker block placed on the patient, serving as a surrogate signal for tumor motion. The CT images were reviewed, and 4D CT data were analyzed by a physicist in GE AW Server 3.2 Ext 4.0 (GE Healthcare, Milwaukee, WI) to determine the appropriate motion control method for a lung SBRT patient. If the target exhibited large motion or was in proximity to OARs, as previously described, a DIBH CT scan would be acquired if the patient could hold his/her breath well.

Effective breath‐holding capability is essential for undergoing DIBH. Prior to the DIBH scan, the patient underwent training to hold his/her breath at deep inspiration multiple times until a steady breath‐hold of at least 15 s was achieved. If a patient was unable to achieve the desired breath‐hold, an alternative motion control method, such as gating, would be employed. All patients in this study had the ability to hold their breath effectively. The DIBH amplitude was set at the level that the patient could tolerate, and the amplitude window was set to 5 mm. The DIBH CT scan was then acquired at the established breath‐hold level.

### 2.3 Treatment planning and delivery

The DIBH CT images were sent to a Varian Eclipse treatment plan system (TPS) (Varian Medical Systems, Palo Alto, CA) for planning. The size of gross tumor volume (GTV) ranged from 0.4 to 29.7 cc. The PTV was derived from GTV with 5 mm margin in anterior‐posterior (AP), right‐left (RL), and 8 mm in superior‐inferior (SI) directions. The SBRT plans were optimized in Eclipse TPS, employing two partial arcs on a Varian TrueBeam machine. The median span of the partial arc was 170 degrees, with the shortest span at 120 degrees and the longest span at 205 degrees. 6MV FFF beams with a maximum dose rate of 1400 MU/min were utilized. The PTV prescription included either 12 Gy × 4 fractions (for 27 patients) or 10 Gy × 5 fractions (for one patient). The goal for the Biologically Effective Dose (BED) of the GTV was set to be no less than 110 Gy, with an α/β ratio of 10 for the tumor. For normal tissue dose constrains, the NCCN Guidelines^19^ were followed, and the Timmerman tables^20^ were used as a reference. Plan evaluation parameters, including conformality, *R*_50%_ (Ratio of prescription isodose volume to the PTV volume), *D*_2cm_ (maximum dose at 2 cm from PTV in any direction), and lung *V*_20Gy_ (percent of lung receiving 20 Gy or more), were in accordance with the RTOG 0915 guidelines.^21^ All SBRT plans were validated with Varian portal dosimetry (Varian Medical Systems, Palo Alto, CA) before being delivered to patients.

Throughout the treatment, patients were positioned and immobilized in the same manner as during the simulation. To enhanced patient compliance with breath‐hold, a Varian visual coach device (VCD) was used. At the first treatment, prior to the treatment, patients underwent practice to ensure they could effectively comply with the established DIBH level. After the patient setup, gated orthogonal KV/CBCT images were utilized for initial patient alignment, followed by gated CBCT for target alignment. If the shift exceeded 1 cm, CBCT would be repeated. Therapists performed the initial target alignment, with physicists checking and adjusting if necessary, and the physician conducted the final check. The treatment was initiated once the physician approved the alignment at the treatment console. Following each treatment delivery, a post‐treatment gated CBCT was obtained to perform a post‐treatment target alignment. To reduce imaging time, either a gated spotlight CBCT with a 200‐degree gantry span or a gated short thorax CBCT with a 140‐degree gantry span was utilized.

### 2.4 Data analysis

The post‐treatment CBCTs were used to quantify the intra‐fractional tumor shift in each direction, that is, AP, RL, and SI directions. To investigate whether the natural free breath motion amplitude influences the intra‐fractional motion during a breath‐hold, patients were separated into two groups based on the initial free breath (FB) planning 4D CT analysis: one with tumor motion no more than 10 mm (FB motion ≤ 10 mm) and the other with tumor motion more than 10 mm (FB motion > 10 mm). The intra‐fractional motion amplitude with DIBH was then compared between these two groups.

These shifts obtained from the post‐treatment CBCTs were then used to calculate the overall group mean error (*M*, representing the mean of all patients’ mean shifts), the systematic error (*Σ*, denoting the standard deviation around group mean error), and the random error (*σ*, defined as the root mean square of the patients’ standard deviations), as described by van Herk et al.^22^ The PTV margin in each direction was calculated by the following formula (1) to achieve that, for 90% of the patient population, the minimum dose to the GTV must be 95% of the nominal dose (i.e., the dose at the specification point) or higher.

(1) PTVmargin=2.5∑+0.7σ

The SBRT plan beam delivery time was effectively reduced by utilizing two partial arcs and the highest available dose rate. However, patients might encounter difficulties in maintaining a breath‐hold for the entire beam delivery. As a result, multiple instances of DIBH might be required within a single beam delivery. The occurrence of beam on‐hold between consecutive DIBHs inevitably extended the overall beam delivery time (*T*_beam_). The actual beam delivery time with DIBH (*T*_beam_DIBH_) was obtained from Aria for each fraction to be compared with the beam delivery time without DIBH (*T*_beam_wo_DIBH_) for the corresponding SBRT plan. *T*_beam_wo_DIBH_ was recorded during the SBRT plan validation process.

## 3 RESULTS

### 3.1 Amplitude of intra‐fractional tumor shifts

A total of 113 post‐treatment CBCTs were acquired to quantify the intra‐fractional tumor motion in the 28 patients. Based on the initial planning 4D CT analysis with free breath, among these 28 lung lesions, 15 had a motion of no more than 10 mm, while 13 had a motion of more than 10 mm with the maximum motion of 52 mm. For the group with FB motion ≤ 10 mm, 61 post‐treatment CBCTs were acquired. The other group had 52 post‐treatment CBCTs.

**FIGURE 1:**
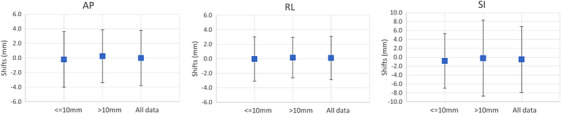
The average shifts with 2 standard deviations for group of FB motion ≤ 10 mm, FB motion > 10 mm, and all shifts.

For the group with FB motion ≤ 10 mm, the average shifts were ‐0.2 ± 1.9 mm, 0.0 ± 1.5 mm, and ‐0.8 ± 3.1 mm in AP, RL, and SI directions, respectively. For the group with FB motion > 10 mm, the average shifts were 0.3 ± 1.8 mm, 0.2 ± 1.4 mm, and −0.2 ± 4.3 mm in AP, RL, and SI directions, respectively. The average shifts from all 113 data were 0.0 ± 1.9 mm, 0.1 ± 1.5 mm, and −0.5 ± 3.7 mm in AP, RL, and SI directions, respectively. Figure 1 shows the average shifts with 2 standard deviations for group of FB motion ≤ 10 mm, FB motion > 10 mm, and all shifts in each direction. In AP and RL directions, the shifts of the two groups were similar. In the SI direction, the group with FB motion > 10 mm displayed a larger shift range than the group with FB motion ≤ 10 mm. However, Mann‐Whitney *U* test indicated no significant differences between the two groups in all directions, with *p*‐values of 0.253, 0.551, and 0.106 in AP, RL, and SI directions, respectively.

**FIGURE 2:**
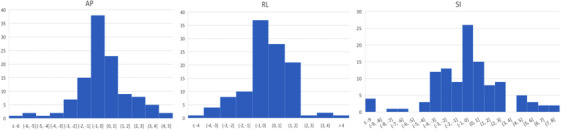
Histogram of intra‐fractional shifts in each direction.

Figure 2 shows the histogram of intra‐fractional shifts in each direction. The range of shifts in SI direction was greater than those in AP and RL directions. The distribution was centered around 0 in each direction. However, the Shapiro‐Francia normality test indicated that the distribution deviated from normality, with *p* < 0.001 in all directions. Nevertheless, Mann‐Whitney *U* test revealed no significant difference between the anterior and posterior shifts, right and left shifts, and superior and inferior shifts, with *p*‐values of 0.337, 0.498, and 0.432 in the AP, RL, and SI directions, respectively.

**FIGURE 3:**
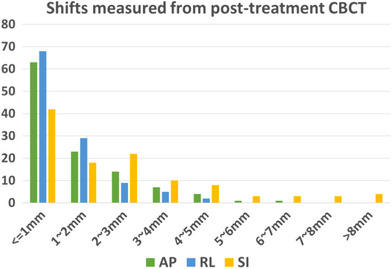
The distribution of absolute intra‐fractional shifts in each direction.

Table 1 lists the percentage of shifts within a threshold. Figure 3 illustrates the distribution of absolute intra‐fractional shifts in each direction. The maximum shifts observed were 6.3 , 5.0 , and 17.7 mm in AP, RL, and SI directions, respectively. At six treatments (5.3%), the shifts exceeded the tolerance defined by the PTV margin. Two shifts exceeded 5 mm in AP direction and four shifts exceeded 8 mm in SI direction.

**TABLE 1:** The percentage of shifts within a threshold.

Threshold	≤ 2 mm	≤ 3 mm	≤ 4 mm	≤ 5 mm	≤ 7 mm	≤ 8 mm
AP	76.1	88.5	94.7	98.2	100.0	100
RL (%)	85.8	93.8	98.2	100.0	100.0	100
SI	53.1	72.6	81.4	88.5	93.8	96.5

Abbreviations: AP, anterior‐posterior; RL, right‐left; SI, superior‐inferior.

### 3.2 PTV margin adequacy

Table 2 shows the calculated overall group mean error (*M*), the systematic error (*Σ*), and the random error (*σ*) in each direction. Although daily pre‐treatment CBCT enhances setup accuracy, errors can still arise due to the accuracy of the treatment machine's isocenter geometry. The Winston‐Lutz test records show that the average maximum isocenter accuracy of our machine is 0.50 ± 0.16 mm. To take a cautious approach, a 1 mm systematic error, Σ_s_, is considered for setup error in margin calculation. Furthermore, the Van Herk's margin design model assumes that there are many fractions for a treatment course, while lung SBRT typically involves only three to five fractions. In this study, the hypofractionation effect was corrected by using an additional systematic error^22^ *Σ*_f_, while $\Sigma f\;=\;\sigma /\sqrt{N}$ with *N* being the number of fractions. The total systematic error, *Σ*_T_, was calculated as $\Sigma_{T}=\sqrt{\Sigma^{2}+\Sigma_{s}^{2}++\;\Sigma_{f}^{2}}\;$_,_ Σ_T _= 1.6, 1.4, and 2.3 mm in AP, RL, and SI directions, respectively.

**TABLE 2:** Overall group mean error (*M*), the systematic error (*Σ*), and the random error (*σ*).

	AP	RL	SI
*M* (mm)	0.1	0.1	‐0.5
*Σ* (mm)	1.2	0.9	1.8
*σ* (mm)	0.7	0.7	2.3

Abbreviations: AP, anterior‐posterior; RL, right‐left; SI, superior‐inferior.

The PTV margin, calculated using formula (1) with correction for SBRT hypofractionation and incorporating setup errors, is determined to be 4.5 , 3.9 , and 7.4 mm in AP, RL, and SI directions, respectively.

### 3.3 Beam delivery time

The average *T*_beam_wo_DIBH_ and *T*_beam_DIBH_ were 2.4 ± 0.4 min and 3.6 ± 1.5 min, respectively. The average treatment slot for lung SBRT with DIBH was 25.3 ± 7.9 min. While the *t*‐tests indicated a significant difference between *T*_beam_wo_DIBH_ and *T*_beam_DIBH_ (*p* value < 0.01), the time increase of *T*_beam_DIBH_, relative to the treatment slot, is not clinically significant.

## 4 DISCUSSION

In our clinic, patients generally exhibit better tolerance for respiratory gating compared to DIBH. This may be attributed to the fact that, with respiratory gating, patients breathe in a manner similar to their natural breath, whether coached or uncoached. Therefore, we utilize DIBH only when it has the potential to reduce treatment time compared to gating or when it offers better sparing of normal tissue or OARs. DIBH is specifically employed for patients who can consistently achieve and maintain the required breath‐hold position after training.

In this study, we measured the intra‐fractional tumor motion by utilizing CBCTs acquired after treatment for patients receiving lung SBRT with visually coached DIBH. The shifts in SI direction were larger than those in AP and RL directions. The shifts in approximately 95% of the treatments fell within the tolerance defined by the current applied PTV margin, which was 5 mm margin in AP and RL directions and 8 mm in SI direction.

We categorized patients into two groups based on the amplitude of tumor motion under free breath: FB motion ≤ 10 mm and FB motion > 10 mm. This categorization aimed to investigate whether larger free breath motion would result in larger or smaller intra‐fractional shifts during DIBH treatment. Our results revealed no significant difference between the two groups. This suggests that the natural free breath motion amplitude does not have, or has very little, influence on intra‐fractional tumor shifts during DIBH treatment.

The overall group mean error (*M*), systematic error (*Σ*), and random error (*σ*) from our study were comparable to the findings of Josipovic et al.^15^ in the AP and RL directions, but notably larger in the SI direction—1.8  versus 1.1 mm for *Σ* and 2.3  versus 1 mm for *σ*. It should be noted that Josipovic et al assessed intra‐fractional tumor motion using three consecutive DIBH CT scans on the day of the planning CT scan. In contrast, our data, obtained from post‐treatment CBCT, is closer to reflecting the actual intra‐fractional motion during treatment. Another possible factor contributing to the difference may be the width setting of the DIBH gating window. Our setting was 5 mm, while theirs ranged from 2.5 to 3 mm. The studies conducted by Peng et al.^16^ and Scherman Rydhög et al.^17^ also demonstrated smaller intra‐fractional motion shifts. Peng et al. employed 5−7 consecutive DIBH planning CT scans, while Scherman Rydhög et al. used perpendicular fluoroscopic movies for their measurements. Ottosson et al.^18^ evaluated intra‐fractional tumor shifts using mid‐treatment CBCT. Their results showed larger values for *M*, *Σ*, and *σ* compared to our results, despite their gating window being set at 2−3 mm. The substantial variation in study results among different institutes, including ours, suggests that intra‐fractional tumor motion during DIBH treatment may vary depending on the implementation procedure and patient population in different institutions.

Recently, ESTRO‐ACROP published a guideline^23^ titled “Recommendations on the Implementation of Breath‐Hold Techniques in Radiotherapy.” One of their recommendations is that each institution allocates time and resources for its own quality assurance program to assess inter‐fraction and, ideally, intra‐fraction uncertainties. 3D Imaging during treatment and/or post‐treatment is preferred to estimate intra‐fractional motion. So far, there is not much data published on this topic from different institutes.

Some studies^24^, ^25^, ^26^ employed surface‐guided radiotherapy (SGRT) to monitor intra‐fractional motion during lung SBRT with DIBH. While SGRT provides real‐time monitoring and guidance for patient positioning during treatment, enhancing setup accuracy, it is important to note that surface‐based systems only capture images of the patient's external surface. The direct correlation to internal anatomy remains uncertain. Therefore, despite the advantages of SGRT, 3D imaging is still required to assess intra‐fractional tumor motion accurately.

The ESTRO‐ACROP guideline also emphasizes that margin reduction should be approached with caution, taking into consideration all uncertainties introduced by the DIBH procedure itself. When designing the PTV margin, it should be cautious about reducing the treatment margin based on improved accuracy in one aspect, such as setup error. Doing so could be risky and may result in a significant underdosage of the GTV/CTV. It is crucial to acknowledge that DIBH does not eliminate but mitigates the target position uncertainty, which needs to be accounted for in treatment margins. Margin design should be based on data from larger representative patient groups. With CBCT‐guided treatment setup and tumor alignment, inter‐fractional motion is corrected; therefore, intra‐fractional motion predominates the PTV margin design. Despite the enhanced setup accuracy provided by daily pre‐treatment CBCT, errors may still occur due to the accuracy of the treatment machine's isocenter geometry. In this study, a setup error of 1 mm was used for margin calculation.

The calculated PTV margin was 4.5 , 3.9 , and 7.4 mm in AP, RL, and SI directions, respectively. These values closely aligned with the shift values corresponding to the 95% threshold in Table 1. For example, in SI direction, 93.8% of shifts ≤ 7 mm and 96.5% of shifts ≤ 8 mm, therefore the threshold for 95% of shifts would fall into the 7−8 mm range. Our current applied PTV margins are 5 mm in AP and RL directions, and 8 mm in SI directions, The study suggests that these margins are adequate for our lung SBRT with DIBH treatment. It is safe to consider reducing the margin in the RL direction to 4 mm to enhance the sparing of surrounding normal tissue or OARs.

When planning for lung SBRT with DIBH, efforts were made to minimize beam delivery time by shortening the partial VMAT arc span, utilizing the maximum dose rate of 1400 MU/min, and restricting the number of MUs. However, most single beam delivery times still exceeded 1 min. In most patients, multiple instances of DIBH were needed within a single beam delivery, resulting in an overall increase in beam delivery time. The average *T*_beam_wo_DIBH_ and *T*_beam_DIBH_ were 2.4 ± 0.4 min and 3.6 ± 1.5 min, respectively. Relative to the treatment slot of 25 min, the increase in beam delivery time was not clinically significant.

## 5 CONCLUSION

Intra‐fractional tumor motion is the predominant source of treatment uncertainties in CBCT‐guided lung SBRT with DIBH. The required PTV margin should be determined based on data specific to each institute, considering different techniques and populations. Our data indicates that our current applied PTV margin is adequate, and there is potential for further reduction, particularly in RL direction.

The beam delivery time was extended with DIBH, approximately 1.5 times that without DIBH. However, relative to the treatment slot of 25 min, the increase in beam delivery time was not clinically significant.

## AUTHOR CONTRIBUTIONS

All authors contributed to this work.

## CONFLICT OF INTEREST STATEMENT

The authors declare no conflicts of interest.

## References

[1] Ball D , Mai GT , Vinod S , et al. Stereotactic ablative radiotherapy versus standard radiotherapy in stage 1 non‐small‐cell lung cancer (TROG 09.02 CHISEL): a phase 3, open‐label, randomised controlled trial. Lancet Oncol. 2019;20(4):494‐503. doi:10.1016/S1470-2045(18)30896-9 30770291

[2] Hansen O , Kristiansen C , Nielsen M , Schytte T , Starup Jeppesen S . Survival after stereotactic radiotherapy in patients with early‐stage non‐small cell lung cancer. Acta Oncol. 2019;58(10):1399‐1403. doi:10.1080/0284186X.2019.1631476 31271094

[3] Chang JY , Mehran RJ , Feng L , et al. Stereotactic ablative radiotherapy for operable stage I non‐small‐cell lung cancer (revised STARS): long‐term results of a single‐arm, prospective trial with prespecified comparison to surgery. Lancet Oncol. 2021;22(10):1448‐1457. doi:10.1016/S1470-2045(21)00401-0 34529930 PMC8521627

[4] Chang JY , Senan S , Paul MA , et al. Stereotactic ablative radiotherapy versus lobectomy for operable stage I non‐small‐cell lung cancer: a pooled analysis of two randomised trials. Lancet Oncol. 2015;16(6):630‐637. doi:10.1016/S1470-2045(15)70168-3 25981812 PMC4489408

[5] Brandner ED , Chetty IJ , Giaddui TG , Xiao Y , Huq MS . Motion management strategies and technical issues associated with stereotactic body radiotherapy of thoracic and upper abdominal tumors: a review from NRG oncology. Med phys. 2017;44(6):2595‐2612. doi:10.1002/mp.12227 28317123 PMC5473359

[6] Keall PJ , Mageras GS , Balter JM , et al. The management of respiratory motion in radiation oncology report of AAPM Task Group 76. Med Phys. 2006;33(10):3874‐3900. doi:10.1118/1.2349696 17089851

[7] Aznar MC , Maraldo MV , Schut DA , et al. Minimizing late effects for patients with mediastinal Hodgkin lymphoma: deep inspiration breath‐hold, IMRT, or both? Int J Radiat Oncol Biol Phys. 2015;92(1):169‐174. doi:10.1016/j.ijrobp.2015.01.013 25754634

[8] Everett AS , Hoppe BS , Louis D , et al. Comparison of techniques for involved‐site radiation therapy in patients with lower mediastinal lymphoma. Pract Radiat Oncol. 2019;9(6):426‐434. doi:10.1016/j.prro.2019.05.009 31128302

[9] Persson GF , Scherman Rydhög J , Josipovic M , et al. Deep inspiration breath‐hold volumetric modulated arc radiotherapy decreases dose to mediastinal structures in locally advanced lung cancer. Acta Oncol. 2016;55(8):1053‐1056. doi:10.3109/0284186X.2016.1142115 26935017

[10] Ottosson W , Rahma F , Sjöström D , Behrens CF , Sibolt P . The advantage of deep‐inspiration breath‐hold and cone‐beam CT based soft‐tissue registration for locally advanced lung cancer radiotherapy DIBH radiotherapy for lung cancer patients. Radiother Oncol. 2016;119(3):432‐437. doi:10.1016/j.radonc.2016.03.012 27072938

[11] Josipovic M , Persson GF , Håkansson K , et al. Deep inspiration breath hold radiotherapy for locally advanced lung cancer: comparison of different treatment techniques on target coverage, lung dose and treatment delivery time. Acta Oncol. 2013;52(7):1582‐1586. doi:10.3109/0284186x.2013.813644 24047341

[12] Marchand V , Zefkili S , Desrousseaux J , Simon L , Dauphinot C , Giraud P . Dosimetric comparison of free‐breathing and deep inspiration breath‐hold radiotherapy for lung cancer. Strahlenther Onkol. 2012;188(7):582‐589. doi:10.1007/s00066-012-0129-9 22588467

[13] Mani KR , , Bhuiyan MA , Alam MM , et al. Dosimetric comparison of deep inspiration breath hold and free breathing technique in stereotactic body radiotherapy for localized lung tumor using flattening filter free beam. Pol J Med Phys Eng. 2018;24(1):15‐24. doi:10.2478/pjmpe-2018-0003

[14] Mørkeset ST , Lervåg C , Lund J‐Å , Jensen C . Clinical experience of volumetric‐modulated flattening filter free stereotactic body radiation therapy of lesions in the lung with deep inspiration breath‐hold. J Appl Clin Med Phys. 2022;23(9):e13733. doi:10.1002/acm2.13733 35867387 PMC9512343

[15] Josipovic M , Aznar MC , Thomsen JB , et al. Deep inspiration breath hold in locally advanced lung cancer radiotherapy: validation of intrafractional geometric uncertainties in the INHALE trial. Br J Radiol. 2019;92(1104):20190569. doi:10.1259/bjr.20190569 31544478 PMC6913352

[16] Peng Y , Vedam S , Chang JY , et al. Implementation of feedback‐guided voluntary breath‐hold gating for cone beam CT‐based stereotactic body radiotherapy. Int J Radiat Oncol Biol Phys. 2011;80(3):909‐917. doi:10.1016/j.ijrobp.2010.08.011 21470784

[17] Scherman Rydhög J , Riisgaard de Blanck S , Josipovic M , et al. Target position uncertainty during visually guided deep inspiration breath‐hold radiotherapy in locally advanced lung cancer. Radiother Oncol. 2017;123(1):78‐84. doi:10.1016/j.radonc.2017.02.003 28245908

[18] Ottosson W , Rand Momsen NC , Fortin Jørgensen S , et al. PD‐0232 large intra‐fractional tumor position variations in deep inspiration breath‐hold lung SBRT. Radiother Oncol. 2022;170:S192‐193. doi:10.1016/s0167-8140(22)02787-6

[19] National Comprehensive Cancer Network . NCCN Clinical Practice Guidelines in Oncology (NCCN Guidelines®) Non‐Small Cell Lung Cancer. https://www.nccn.org/guidelines/guidelines‐detail?category=1&id=1450

[20] Timmerman R . A story of hypofractionation and the table on the wall. Int J Radiat Oncol Biol Phys. 2022;112(1):4‐21. doi:10.1016/j.ijrobp.2021.09.027 34919882

[21] RADIATION THERAPY ONCOLOGY GROUP RTOG 0915 (NCCTG N0927) .A Randomized Phase II Study Comparing 2 Stereotactic Body Radiation Therapy (SBRT) Schedules for Medically Inoperable Patients with Stage I Peripheral Non‐Small Cell Lung Cancer SCHEMA. https://pps4rt.com/wp‐content/uploads/2019/07/RTOG‐0915.pdf

[22] Van Herk M , Remeijer P , Rasch C , et al. The probability of correct target dosage:dose‐population histograms for deriving treatment margins in radiotherapy. Int J Radiat Oncol Biol Phys. 2000;47(4):1121‐1135. doi:10.1016/s0360-3016(00)00518-6 10863086

[23] Aznar MC , Corradini S , Mast M , et al. ESTRO‐ACROP guideline: recommendations on implementation of breath‐hold techniques in radiotherapy. Radiother Oncol. 2023;185:109734. doi:10.1016/j.radonc.2023.109734 37301263

[24] Naumann P , Batista V , Farnia B , et al. Feasibility of optical surface‐guidance for position verification and monitoring of stereotactic body radiotherapy in deep‐inspiration breath‐hold. Front Oncol. 2020;10:573279. doi:10.3389/fonc.2020.573279 33102232 PMC7546313

[25] Guo HL , Wu WW , Huan Y , Zhang HW . SGRT‐based stereotactic body radiotherapy for lung cancer setup accuracy and margin of the PTV. J Appl Clin Med Phys. 2024;25:e14195. doi:10.1002/acm2.14195 37915300 PMC10930008

[26] Nguyen D , Reinoso R , Farah J , et al. Reproducibility of surface‐based deep inspiration breath‐hold technique for lung stereotactic body radiotherapy on a closed‐bore gantry linac. Phys Imaging Radiat Oncol. 2023;26:100448. doi:10.1016/j.phro.2023.100448 37252251 PMC10213090

